# Ginsenoside Rg3 inhibits osteosarcoma progression by reducing circ_0003074 expression in a miR-516b-5p/KPNA4-dependent manner

**DOI:** 10.1186/s13018-021-02868-7

**Published:** 2021-12-20

**Authors:** Tehasi Wang, Chengguang Zhang, Shuren Wang

**Affiliations:** 1 Graduate School, Heilongjiang University of Traditional Chinese Medicine, Harbin, Heilongjiang China; 2 Department of Tramotology and Orthopedics, First Affiliated Hospital, Heilongjiang University of Chinese Medicine, No. 26 Heping Road, Xiangfang District, Harbin, 150040 Heilongjiang China

**Keywords:** Rg3, OS, circ_0003074, miR-516b-5p, KPNA4

## Abstract

**Background:**

Previous data have suggested that ginsenoside Rg3 (Rg3), isolated from the roots of Panax ginseng, plays a repressing role in multiple cancers, including osteosarcoma (OS). However, there is no any literature available about the role of circular RNA (circRNA) in Rg3-mediated OS development. The study aimed to explore the function of circ_0003074 in the anti-cancer effects of Rg3 on OS.

**Methods:**

RNA expression of circ_0003074, miR-516b-5p and karyopherin subunit alpha 4 (KPNA4) was detected by quantitative real-time polymerase chain reaction (qRT-PCR). Protein expression was evaluated by Western blotting or immunohistochemistry assay. Cell viability, proliferation, apoptosis, migration and invasion were investigated by cell counting kit-8, 5-ethynyl-29-deoxyuridine (EdU), flow cytometry analysis, wound-healing and transwell invasion assays, respectively. Dual-luciferase reporter and/or RNA immunoprecipitation assay was performed to confirm the interplay between miR-516b-5p and circ_0003074 or KPNA4. Xenograft mouse model assay was conducted to reveal the effect of Rg3 treatment on tumor formation.

**Results:**

Circ_0003074 and KPNA4 expression was significantly upregulated, while miR-516b-5p was downregulated in OS tissues and cells compared with controls. Rg3 treatment dramatically decreased circ_0003074 expression in OS cells. Rg3 treatment led to decreased cell proliferation, migration and invasion but increased cell apoptosis, which was attenuated after circ_0003074 overexpression. Besides, miR-516b-5p was a target miRNA of circ_0003074 and partially restored circ_0003074-mediated action under Rg3 treatment. Decreasing miR-516b-5p expression also promoted Rg3-treated OS cell malignancy through KPNA4, which was identified as a target mRNA of miR-516b-5p. Besides, circ_0003074 induced KPNA4 production owing to the decrease of miR-516b-5p expression. Furthermore, Rg3 treatment inhibited tumor formation by regulating circ_0003074 in vivo.

**Conclusion:**

Rg3 inhibited OS progression through circ_0003074/miR-516b-5p/KPNA4 axis, showing the potential of Rg3 as a therapeutic agent for OS.

**Supplementary Information:**

The online version contains supplementary material available at 10.1186/s13018-021-02868-7.

## Abstract (highlights)

1. Circ_0003074 expression was upregulated in OS tissues and cells.
2. Rg3 treatment significantly decreased circ_0003074 expression in OS cells.
3. Circ_0003074 overexpression rescued Rg3-induced inhibition in OS progression.
4. Circ_0003074 induced KPNA4 production through miR-516b-5p under Rg3 treatment.

**Supplementary Information:**

The online version contains supplementary material available at 10.1186/s13018-021-02868-7.

## Introduction

As a primary bone malignancy that is common in children as well as adolescents, osteosarcoma (OS) originates from mesenchymal cells with high mortality and disability [1]. Despite significant development in the combined treatment of surgical technology and chemotherapy, the long-term survival rate of OS cases is still low owing to metastasis and recurrence [2]. As a result, developing a new drug is necessary to improve the poor clinical outcome. Ginsenoside Rg3 (Rg3) is a relatively safe active component that is extracted from ginseng [3]. Rg3 has been involved in the regulation of some types of cancers like colorectal cancer [4], breast cancer [5] and thyroid cancer [6]. In particular, Rg3 inhibits OS cell proliferation and induces cell apoptosis [7, 8], and considerable evidence has suggested that graphene oxide nanoparticle-loaded RG3 inhibits OS cell malignancy [9], showing the potential of Rg3 as a therapeutic agent for OS. Exploring the inner mechanism regarding Rg3-mediated OS cell malignancy is helpful to develop Rg3 as a therapeutic agent of the disease.

Circular RNA (circRNA) is an endogenous RNA consisted of a closed covalent loop with 5' or 3' polarities structure, which is formed by head-to-tail splicing [10]. CircRNA functions by interacting with RNA-binding protein, acting as a microRNA (miRNA) sponge, or serving as a transcription regulator [11]. Previous study showed that circRNA played important roles in OS progression [12]; however, no circRNA has been identified as the target of Rg3. Circ_0003074, located in chr10:12123470–12133683, is formed by circulation of dehydrogenase E1 and transketolase domain containing 1 (DHTKD1) previous data have suggested that circ_0003074 is the most significantly upregulated one in OS tissues in comparison with adjacent tissues among the 20 upregulated circRNA, and its expression is associated with clinical characteristics of OS patients [13]; however, there is no study involving whether the circRNA participates in Rg3-mediated OS malignant progression.

As a noncoding regulator, miRNA is composed of 18–25 nucleotides and recognizes target mRNA through base pairing with the noncoding sequence of the gene, so as to post-transcriptionally regulate mRNA expression. MiRNA plays important parts in the pathogenesis of the musculoskeletal system, and previous study indicates the potential of miRNA as the therapeutic target of tendon healing [14]. Besides, miRNA participates in osteoarthritis progression through regulation toward multiple biological processes, such as inflammatory response, chondrogenic differentiation, bone development and homeostasis as well as osteogenic differentiation [15]. The small regulator is involved in the regulation of OS cell processes, which contain cell growth, metastasis, autonomy and apoptosis, mainly by Notch, Wnt, NF-κB, p53 and/or MAPK signaling [16]. Current evidence has indicated that the behind mechanism related to the effects of Rg3 on cancer cell processes involves miRNAs, such as miR-4425 [17] and miR-532-3p [18]. Through online database prediction, we found that miR-516b-5p was a potential target miRNA of circ_0003074. Also, in preliminary experiments, Rg3 treatment downregulated circ_0003074 but upregulated miR-516b-5p in a dose-dependent manner. Importantly, miR-516b-5p has been demonstrated to inhibit OS cell growth by binding to transcription effector Gli1 [19].

Thus, we hypothesized that the mechanism of Rg3 in regulating OS development involved circ_0003074 and miR-516b-5p. The study was designed to determine whether Rg3 could regulate OS cell malignancy through circ_0003074/miR-516b-5p pathway and to explore the downstream target of circ_0003074/miR-516b-5p axis in regulating RG3-mediated OS progression. Herein, we found that Rg3 treatment inhibited OS cell proliferation, migration and invasion but induced cell apoptosis. Besides, circ_0003074 overexpression counteracted Rg3-induced effects in OS cells. Further, circ_0003074/miR-516b-5p/karyopherin subunit alpha 4 (KPNA4) pathway participated in Rg3-mediated OS cell malignancy.

## Materials and methods

### Patients and specimens

Thirty-eight OS patients who underwent complete resection in Heilongjiang University of Traditional Chinese Medicine were recruited for the collection of conventional OS tissues and neighboring non-tumor bone tissues (> 3 cm from the matched OS specimen). No anticancer treatment was performed on these patients prior to operation. All participants provided the written informed consent. Tissues were immediately stored at − 80 °C. The study was approved by the Ethics Committee of Heilongjiang University of Traditional Chinese Medicine.

### Cell culture and treatment

Procell (Wuhan, China) provided OS cell-line MG63 and Saos2, and human normal osteoblast cell-line hFOB1.19, which were cultured in Dulbecco’s modified Eagle’s medium (DMEM; Gibco, Carlsbad, CA, USA) or McCoy’s 5a medium (Gibco) added with fetal bovine serum (FBS; Biosun, Shanghai, China) and 1% penicillin/streptomycin (Gibco) in humid incubators containing 5% CO_2_. hFOB1.19 cells were cultured at 34 °C, and other types of cells were grown at 37 °C.

To screen the suitable time of Rg3 in inhibiting MG63 and Saos2 cell dysfunction caused by OS, the two types of cells were treated with Rg3 at a dose of 0, 40, 80 or 120 µM for 24, 48 or 72 h. To determine the effects of Rg3 on gene expression and OS cell processes, MG63 and Saos2 cells were treated with Rg3 at a dose of 0, 40, 80 or 120 µM for 48 h. In order to reveal the mechanism responsible for Rg3-mediated OS cell malignancy, MG63 and Saos2 cells were treated with 80 µM Rg3 for 48 h.

### Cell transfection

GenePharma (Shanghai, China) synthesized miR-516b-5p mimics (miR-516b-5p, 5ʹ-AUCUGGAGGUAAGAAGCACUUU-3ʹ), miR-516b-5p inhibitors (anti-miR-516b-5p, 5ʹ-AAAGUGCUUCUUACCUCCAGAU-3ʹ), the small interfering RNA (siRNA) for KPNA4 (si-KPNA4, 5ʹ-CAGTGATCGAAATCCACCAATTGAT-3ʹ) and matched controls (miR-NC, anti-miR-NC and si-NC). The plasmid upregulating circ_0003074 expression in OS cells was constructed by introducing full-length circ_0003074 (1005 bp) into pCD5-ciR vector (pCD-ciR; Geneseed, Guangzhou, China), named as circ_0003074. In accordance with the manufacturer’s recommendations of Polyplus-transfection® (Illkirch, France), mimics, inhibitors, siRNA, plasmids and matched controls were transfected into MG63 and Saos2 cells.

### Quantitative real-time polymerase chain reaction (qRT-PCR)

TRIzol kit (Invitrogen, Carlsbad, CA, USA) and miRNA Purification kit (CWBIO, Beijing, China) were used to isolate RNA and miRNA, respectively, from clinical specimens and cells. The extracted RNA was used for synthesizing cDNA using FastKing RT reagents (Tiangen, Beijing, China). PrimeScript miRNA reverse transcription reagents (TaKaRa, Dalian, China) were adopted for reverse transcription of miRNA. qRT-PCR reaction was performed on a qRT-PCR system (Bio-Rad, Hercules, CA, USA) after mixing of SYBR Green Premix (TaKaRa), primers (shown in Table 1) and cDNA/miRNA. The relative expression of circRNA/miRNA/mRNA was analyzed by the 2^−∆∆Ct^ method with the normalization to U6 or glyceraldehyde 3-phosphate dehydrogenase (GAPDH).

**Table 1:** Primers sequences used for qRT-PCR

Name	Sequence (5ʹ-3ʹ)
*hsa_circ_0003074*	
Forward	CAGAATTGGTGGGAGTGTGC
Reverse	GGCCATGATCAACAATATCACTGC
*DHTKD1*	
Forward	CCTCCAGAGCTGATGTTCCG
Reverse	AGAGTAATCGCCGTCTTGGC
*KPNA4*	
Forward	GAATGTGGAGGGCTGGAGAA
Reverse	GCCTCTGGAACAAGGCTAGG
*miR-516b-5p*	
Forward	GCCGAGATCTGGAGGTAAGAAG
Reverse	CTCAACTGGTGTCGTGGAGT
*GAPDH*	
Forward	GGAGCGAGATCCCTCCAAAAT
Reverse	GGCTGTTGTCATACTTCTCATGG
*U6*	
Forward	CTCGCTTCGGCAGCACA
Reverse	AACGCTTCACGAATTTGCGT

DHTKD1: dehydrogenase E1 and transketolase domain containing 1

### Stability analysis of circ_0003074

The stability of circ_0003074 was analyzed using RNase R (ZhongBeiLinGe Biotechnology, Beijing, China) as well as Actinomycin D (Amresco, Solon, OH, USA). One μg RNA from MG63 and Saos2 cells was pre-incubated with 4 U RNase R in an incubator to digest linear RNA. Additionally, MG63 and Saos2 cells were allowed to grow in the media supplied with 2 μg/mL Actinomycin D for 0, 12, 24 and 36 h, followed by RNA isolation according to the aforementioned method. At last, RNA was subjected to qRT-PCR analysis to confirm circ_0003074 and DHTKD1 relative expression.

### Cell viability

The assay regarding cell counting kit-8 (CCK-8) reagent (Dojindo, Shanghai, China) was implemented to analyze cell viability. Briefly, MG63 and Saos2 cells were treated with Rg3, circ_0003074, miR-516b-5p, anti-miR-516b-5p, si-KPNA4 and respective controls according to the defined purpose. At the defined time (24, 48 or 72 h), medium supernatant was removed and cells were incubated with CCK-8 reagent as per guidebook. At last, enzyme immunoassay analyzer (BioTek, Winooski, VT, USA) was employed to analyze samples with absorbance at 450 nm.

### Cell proliferation

MG63 and Saos2 cells were allowed to grow in 6-well plates prior to treatment of Rg3, plasmids or oligonucleotides. At 48 h after transfection, cells were digested and added into each well of 96-well plates, which were supplemented with EdU labeling medium. Subsequent steps were carried out following the user’s manual of EdU staining reagents (Ribobio, Guangzhou, China). Finally, confocal microscope (Olympus, Tokyo, Japan) was applied to determine EdU-positive cells.

### Flow cytometry analysis for cell apoptosis

Treated MG63 and Saos2 cells were harvested by centrifuging and washed using phosphate buffer solution (PBS). The following procedures were performed with an Annexin V-FITC apoptosis detection kit (Solarbio, Beijing, China). In short, these collected cells were suspended in Binding Buffer, followed by incubating with Annexin V-FITC and propidium iodide. Apoptotic cells were analyzed using a flow cytometer (Thermo Fisher, Waltham, MA, USA).

### Wound-healing assay

MG63 and Saos2 cells at ~ 70% confluence were treated with test compounds and cultured in 6-well plates for wound-healing assay. The single-cell layer was scratched using fine pipette tips, and cells were allowed to grow for 24 h in the serum-free McCoy’s 5a (Gibco) or DMEM medium (Gibco). For determining migratory ability of OS cells, the rate of wound closure was observed with a microscope (100 × magnification, Olympus).

### Tranwell invasion assay

Matrigel invasion compartments (Costar, Shanghai, China) were used to analyze cell invasive ability following the guidebook. In brief, the cells treated with test compounds were passaged in the upper chamber, which contained serum-free McCoy’s 5a (Gibco) or DMEM medium (Gibco), followed by 24-h culture. After fixing using methanol (Seebio Biotech, Shanghai, China) and staining using crystal violet (Seebio Biotech), these cells invaded into the lower chambers were counted in a microscope (100 × magnification).

### Western blotting analysis for protein expression

The lysates from MG63 and Saos2 cells and clinical OS specimens were prepared using total protein extraction reagent (Phygene, Fuzhou, China), loaded on 4–12% SurePAGE gels and electrotransferred onto polyvinylidene fluoride membranes. 5% fat-free milk was then employed to block aspecific signals on these membranes. Afterward, the primary antibody specific to proliferating cell nuclear antigen (PCNA; Cat##13-3940; 1:1000; Thermo Fisher), cleaved caspase-3 (Cat #PA5-17913; 1:1000; Thermo Fisher), matrix metalloprotein 9 (MMP9; Cat #PA5-13199; 1:2000; Thermo Fisher), KPNA4 (Cat #PA5-21749; 1:2000; Thermo Fisher) or GAPDH (Cat #MA1-16757; 1:2000; Thermo Fisher) was adopted to incubate these membranes. Immunocomplexes were visualized using RapidStep ECL Reagent (Millipore, Bradford, MA, USA). Secondary antibodies were purchased from Thermo Fisher and used at a dilution of 1:5000–1:15,000.

### Dual-luciferase reporter assay

Online database starbase (http://starbase.sysu.edu.cn/agoClipRNA.php?source=mRNA) was employed to predict the complementary sites of miR-516b-5p with circ_0003074 and the 3ʹ-untranslated region (3ʹUTR) of KPNA4. The sequences of circ_0003074 and KPNA4 3ʹUTR with or without the binding sites of miR-516b-5p were used to build the wild-type (wt) or mutant (mut) plasmids, which included wt-circ_0003074, mut-circ_0003074, wt-KPNA4 3ʹUTR and mut-KPNA4 3ʹUTR. Then, plasmids were transfected into both MG63 and Saos2 cells with miR-516b-5p or miR-NC according to the method as shown above. At 48 h after transfection, the binding intensity was determined by a Dual-Lucy Assay Kit (Vazyme, Jiangsu, China).

### RNA immunoprecipitation (RIP) assay

Both MG63 and Saos2 cells were allowed to grow in 12-well plates lasting 18 h and transfected with miR-516b-5p mimics and matched control (miR-NC). At 48 h post-transfection, AGO2 or IgG-combined circ_0003074 was enriched inferring to the manufacturer’s direction of Magna RNA immunoprecipitation kit (Millipore). Finally, qRT-PCR was conducted to analyze immunoprecipitated RNA.

### Xenograft mouse model assay

Five-week-old male BALB/c nude mice (*N* = 12) were purchased from Charles River (Beijing, China). MG63 cells were diluted to 2.5 × 10^7^ cells/mL using PBS, and 0.2 mL cell suspension was injected subcutaneously into these nude mice. On the seventh day after MG63 cell injection, each mouse was administered at 20 mg/kg Rg3 daily. After 21 days, all mice were euthanatized with xylazine, followed by collecting the forming tumors. The Animal Care and Use Committee of Heilongjiang University of Traditional Chinese Medicine approved this study.

### Immunohistochemistry (IHC) assay

The tissue from xenograft mouse model assay was embedded into paraffin and dewaxed using xylene. The sections were rehydrated with different concentrations of ethanol solutions prior to performing antigen retrieval. After immersed in H2O2 for 10 min, these sections were incubated with the antibody for Ki-67 (Cat#14-5698-82; 1:100; Thermo Fisher) or Vimentin (Cat #PA5-27231; 1:500; Thermo Fisher). The subsequent steps were implemented inferring to the guidebook of IHC assay kit (Phygene). The stained tissues were captured using a confocal microscope (Olympus).

### Statistical analysis

Data analysis was performed on GraphPad Prism or ImageJ software, and results were shown as means ± standard deviations. The disparities were compared using Spearman’s correlation test, Wilcoxon signed-rank test, Student’s *t* tests or analysis of variance. *P* < 0.05 indicated statistical significance.

## Results

### Rg3 treatment downregulated circ_0003074 expression in MG63 and Saos2 cells

**Fig. 1:**
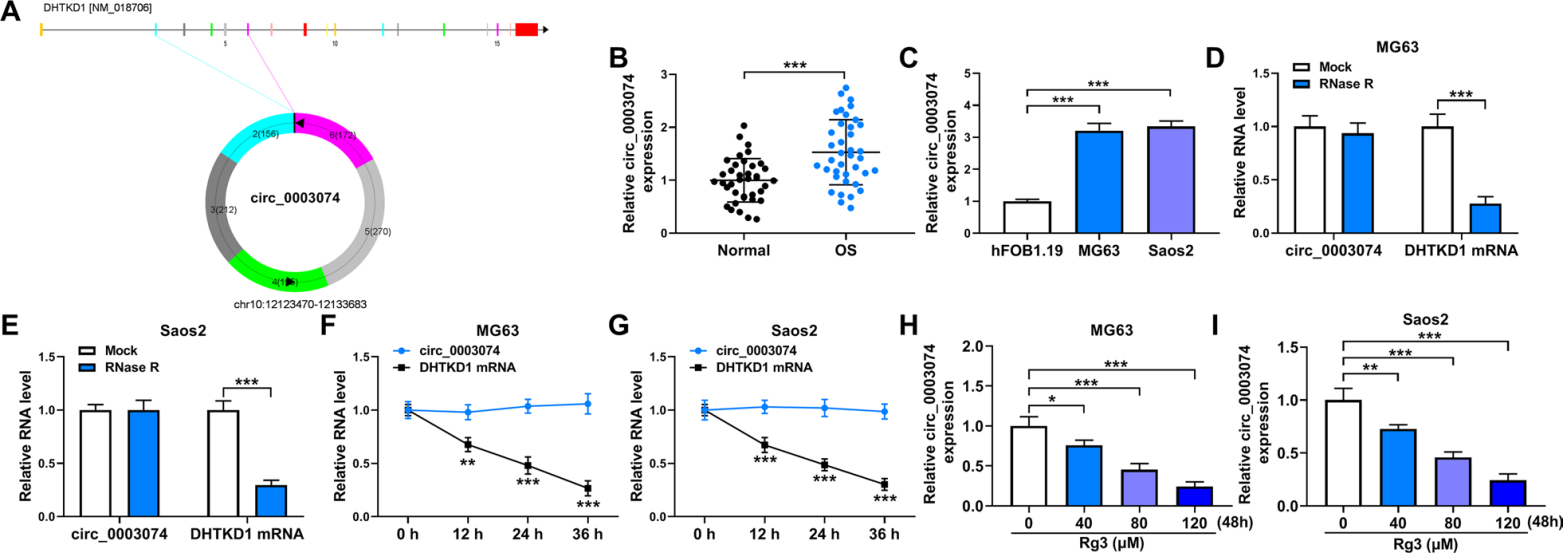
The expression of circ_0003074 in Rg3-treated MG63 and Saos2 cells. **A** The schematic illustration showing the generation of circ_0003074. **B**, **C** Circ_0003074 expression was detected by qRT-PCR in OS tissues (*N* = 38), adjacent normal tissues (*N* = 38), hFOB1.19 cells, MG63 cells and Saos2 cells. **D**–**G** Circ_0003074 stability was identified by RNase R and Actinomycin D treatment assays. **H**, **I** Circ_0003074 expression was determined by qRT-PCR in OS cells treated with Rg3 (0, 40, 80 and 120 µM). *P < 0.05, **P < 0.01 and ***P < 0.001

Circ_0003074 was generated by the cyclization of exons 2–6 of parental DHTKD1 (Fig. 1A), and its expression was first detected by qRT-PCR in OS tissues. As shown in Fig. 1B, circ_0003074 expression was considerably increased in OS tissues in comparison with adjacent normal tissues. Also, OS cell lines (MG63 and Saos2) displayed the high expression of circ_0003074 when compared with normal osteoblast cell line (hFOB1.19) (Fig. 1C). Besides, DHTKD1 expression was dramatically reduced under RNase R or Actinomycin D treatments, but there was no significant difference in circ_0003074 expression (Fig. 1D–G), showing the high stability of circ_0003074. Then, we treated both the MG63 and Saos2 cells with various doses of Rg3 for 24, 48 and 72 h to screen the suitable time of Rg3 in inducing OS cell disorders caused by OS, so as to investigate circ_0003074 expression. As presented in Additional file 1: Figure S1A and B, Rg3 dose and time-dependently inhibited OS cell activity. Given that 48-h treatment of 80 µM Rg3 induced 50% cell viability inhibition, this time point was employed for subsequent study. Consistently, the study found that Rg3 dose-dependently decreased circ_0003074 expression (Fig. 1H and I). Collectively, these data suggested that circ_0003074 might be associated with OS progression.

### Rg3 treatment inhibited OS cell malignancy

**Fig. 2:**
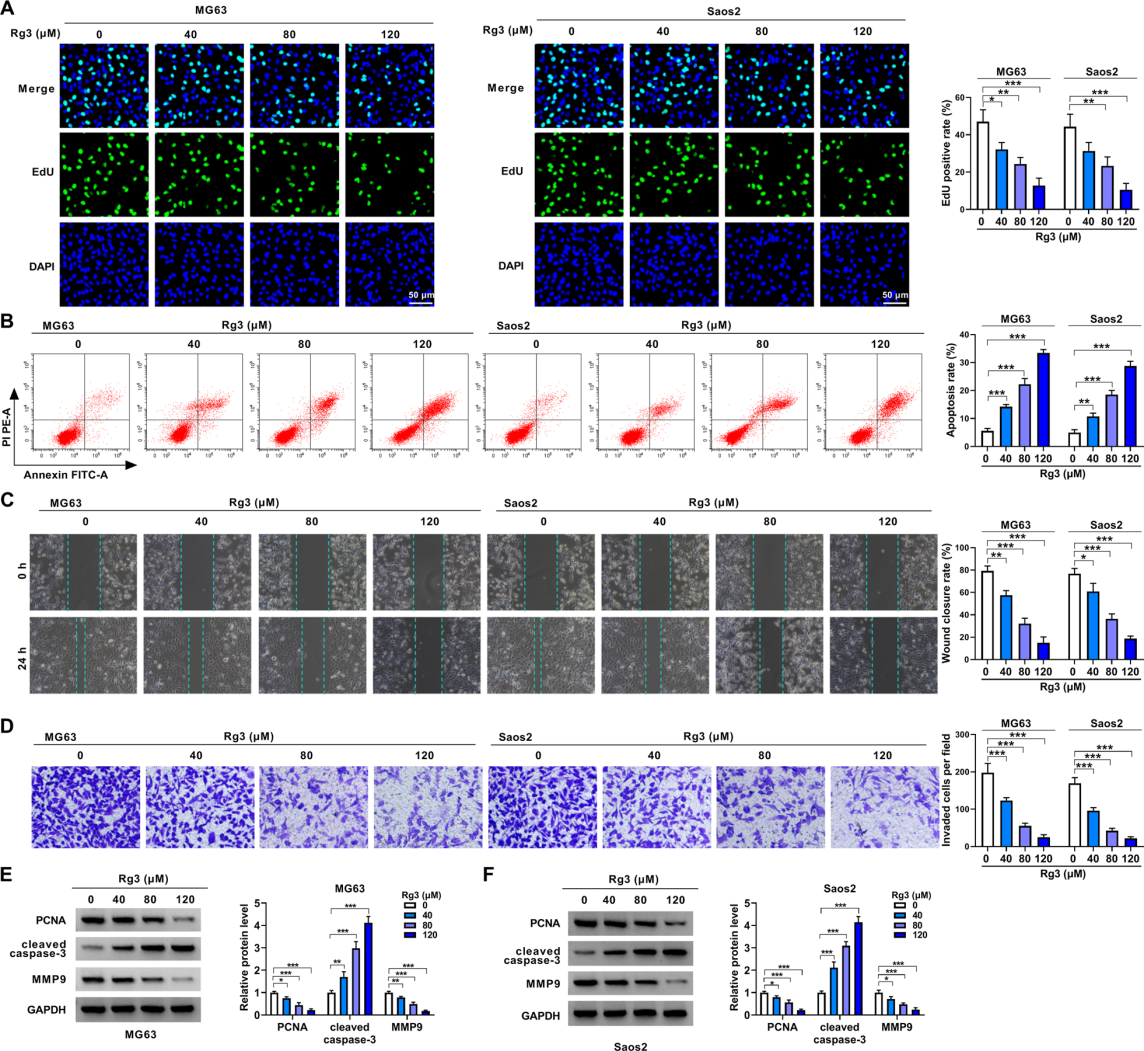
The effects of Rg3 on OS cell malignancy in vitro. **A**–**F** Both MG63 cells and Saos2 cells were treated with Rg3 at a dose of 0, 40, 80 and 120 µM for 48 h and cell proliferation by EdU assay (**A**), cell apoptosis by flow cytometry analysis (**B**), cell migration by wound-healing assay (**C**), cell invasion by transwell invasion assay (**D**) and the protein expression of PCNA, cleaved caspase-3 and MMP9 by Western blotting (**E**, **F**). *P < 0.05, **P < 0.01 and ***P < 0.001

Both MG63 cells and Saos2 cells were treated with various doses of Rg3 for 48 h to determine the consequential effects on cell proliferation, apoptosis, migration and invasion. As expected, Rg3 treatment dose-dependently inhibited cell proliferation (Fig. 2A). Consistently, Rg3 stimulation induced cell apoptosis but inhibited cell migration and invasion in a dose-dependent manner (Fig. 2B–D). Furthermore, we detected the protein expression of proliferation-related PCNA, apoptosis-related cleaved caspase-3 and metastasis-linked MMP9 in the both MG63 cells and Saos2 cells. As a result, we observed decreased PCNA and MMP9 expression but increased cleaved caspase-3 in the two types of cells (Fig. 2E and F). 80 µM Rg3 was selected in the following study as the 50% cell viability inhibition under this condition. Collectively, these findings demonstrated that Rg3 inhibited OS malignant progression in vitro.

### Circ_0003074 overexpression counteracted RG3-induced inhibition of OS cell malignancy

**Fig. 3:**
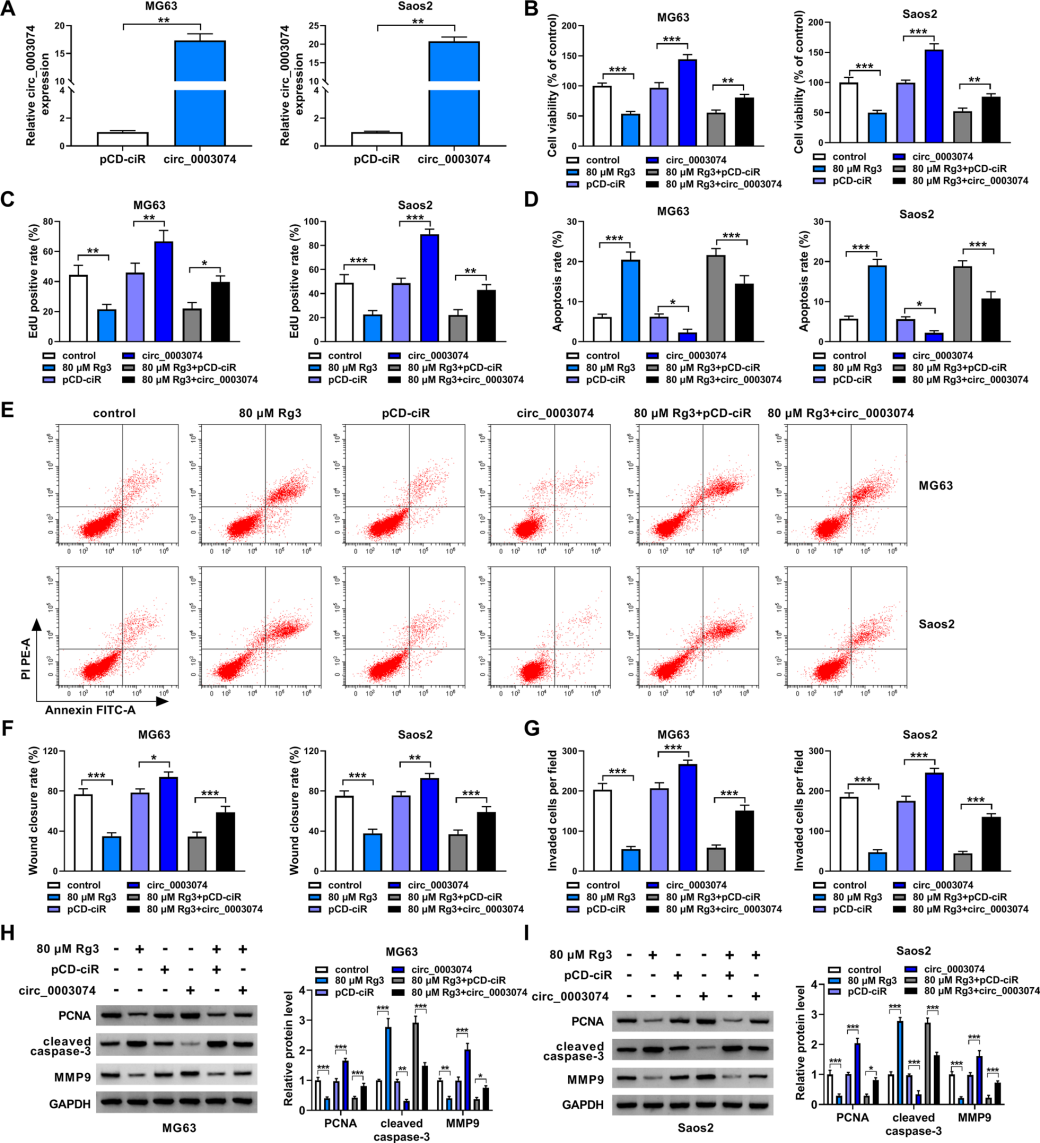
The effects between Rg3 and circ_0003074 overexpression on OS cell malignancy. **A** The efficiency of circ_0003074 overexpression was determined by qRT-PCR. **B**–**I** Both MG63 cells and Saos2 cells were treated with 80 µM Rg3, pCD-ciR, circ_0003074, 80 µM Rg3 + pCD-ciR or 80 µM Rg3 + circ_0003074, with untreated cells as a control, and cell viability was investigated by CCK-8 (**B**), cell proliferation by EdU assay (**C**), cell apoptosis by flow cytometry analysis (**D,** **E**), cell migration by wound-healing assay (**F**), cell invasion by transwell invasion assay (**G**), and the protein expression of PCNA, cleaved caspase-3 and MMP9 by Western blotting (**H,** **I**). *P < 0.05, **P < 0.01 and ***P < 0.001

Based the above results, we continued to explore whether circ_0003074 was involved in Rg3-mediated effects in both MG63 cells and Saos2 cells. Fig. 3A first shows the success of circ_0003074 overexpression in both MG63 cells and Saos2 cells. Then, the study reported that circ_0003074 overexpression promoted cell viability and cell proliferation and relieved the inhibitory effects of Rg3 treatment on cell viability and cell proliferation (Fig. 3B and C). As exhibited in Fig. 3D–G, enforced expression of circ_0003074 inhibited cell apoptosis, promoted cell migration and invasion and attenuated Rg3-induced increase of apoptosis and decreases of cell migration and invasion. Similarly, PCNA and MMP9 protein expression was promoted, cleaved caspase-3 expression was inhibited, and the dysregulated expression of the three proteins induced by Rg3 was relieved when circ_0003074 was overexpressed (Fig. 3H and I). In a word, all evidences confirmed that the inhibitory effects of Rg3 on OS malignant progression in vitro involved the downregulation of circ_0003074.

### Circ_0003074 effectively sponged miR-516b-5p in both MG63 cells and Saos2 cells

**Fig. 4:**
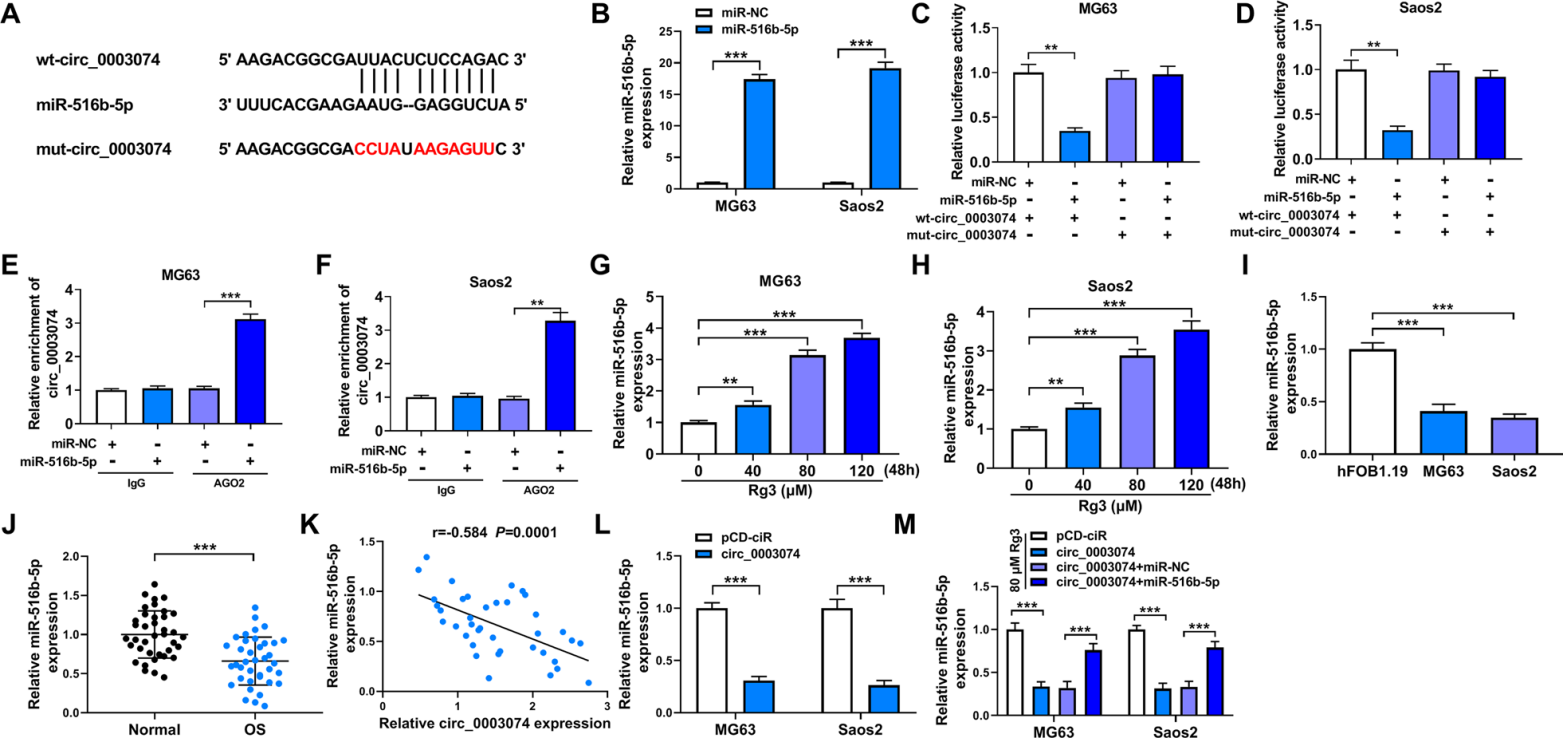
Circ_0003074 combined with miR-516b-5p. **A** The schematic illustration showing the complementary sites of circ_0003074 with miR-516b-5p. **B** The efficiency of miR-516b-5p overexpression was determined by qRT-PCR. **C**–**F** Dual-luciferase reporter and RNA pull-down assays were employed to verify the interplay between circ_0003074 and miR-516b-5p. **G**, **H** MiR-516b-5p expression was detected by qRT-PCR in the both MG63 cells and Saos2 cells treated with Rg3 at a dose of 0, 40, 80 or 120 µM. **I**, **J** MiR-516b-5p expression was checked by qRT-PCR in hFOB1.19 cells, MG63 cells, Saos2 cells, OS tissues (*N* = 38) and adjacent normal tissues (*N* = 38). **K** Spearman correlation analysis was used to predict the correlation of circ_0003074 and miR-516b-5p in OS tissues. **L** The effect of circ_0003074 overexpression on miR-516b-5p expression was revealed by qRT-PCR. **M** qRT-PCR was performed to determine the impact of miR-516b-5p on circ_0003074-induced downregulation of miR-516b-5p. **P < 0.01 and ***P < 0.001

Starbase online database was used to predict the target miRNA of circ_0003074. As presented in Fig. 4A, the seed sequence of miR-516b-5p contained the complementary sites of circ_0003074. Then, dual-luciferase reporter assay was used to testify the binding relationship between circ_0003074 and miR-516b-5p. Figure 4B showed the success of miR-516b-5p overexpression. The study subsequently displayed that increased expression of miR-516b-5p showed a significant inhibition in the luciferase activity of wild-type reporter plasmid of circ_0003074 rather than that of mutant (Fig. 4C and D). Also, the antibody for AGO2 could dramatically enrich circ_0003074 in miR-516b-5p group, as compared with controls (Fig. 4E and F). Thus, the above findings demonstrated that circ_0003074 bound to miR-516b-5p. Besides, we found that Rg3 treatment dose-dependently increased miR-516b-5p expression in both MG63 cells and Saos2 cells (Fig. 4G and H). Comparatively, the miRNA was downregulated in OS cell lines (MG63 cells and Saos2 cells) and OS tissues (Fig. 4I and J). Consistently, we observed the negative correlation of miR-516b-5p and circ_0003074 in expression in OS tissues (Fig. 4K). In support, circ_0003074 negatively regulated miR-516b-5p expression (Fig. 4L). Furthermore, the inhibitory impact of circ_0003074 overexpression on miR-516b-5p expression was relieved after transfection with miR-516b-5p mimics (Fig. 4M).

### Circ_0003074 regulated OS cell malignancy by binding to miR-516b-5p under Rg3 treatment

**Fig. 5:**
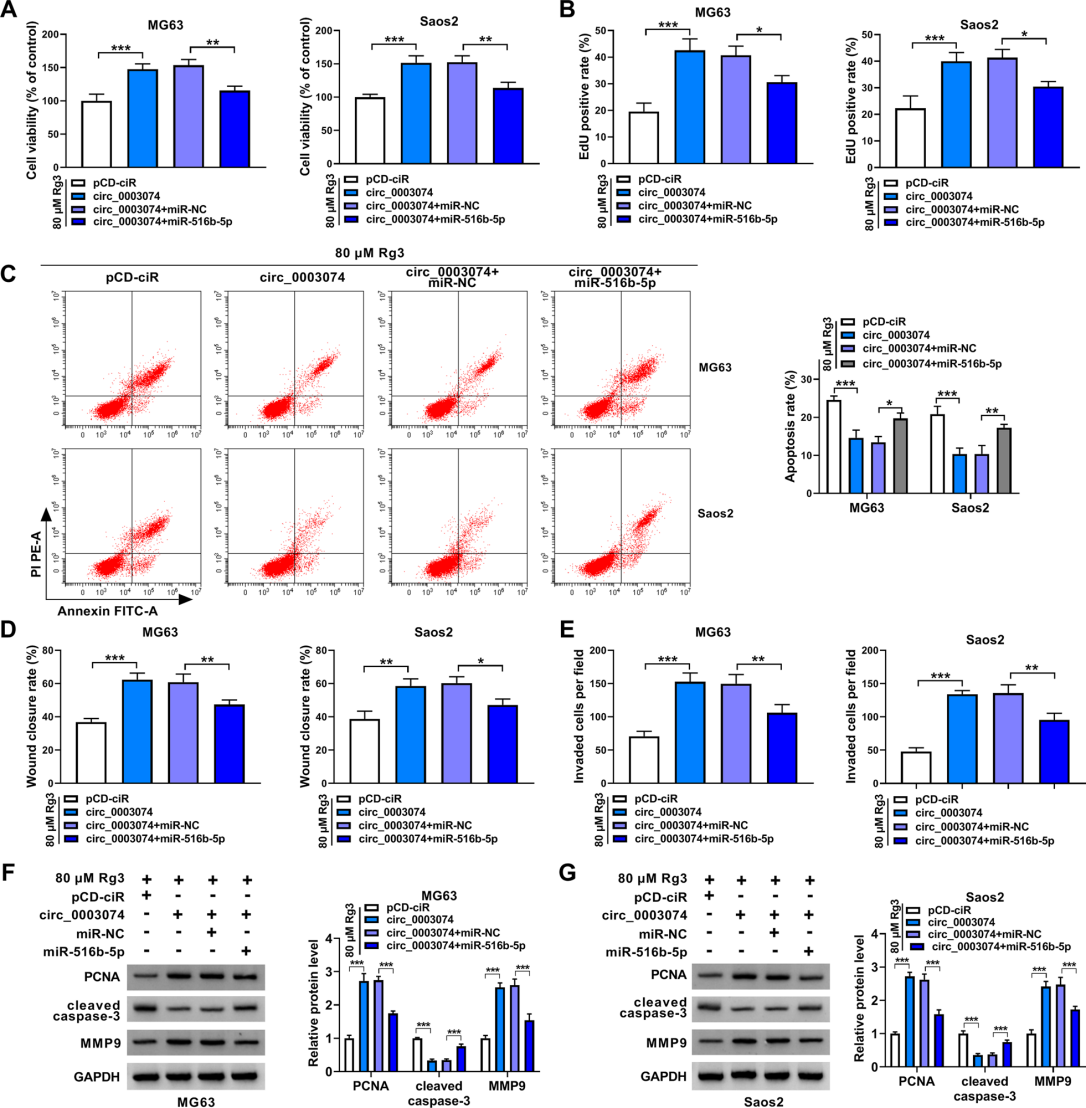
The effects between circ_0003074 and miR-516b-5p on the proliferation, apoptosis, migration and invasion of Rg3-treated MG63 cells and Saos2 cells. **A**–**G** Rg3-treated MG63 cells and Saos2 cells were transfected with pCD-ciR, circ_0003074, circ_0003074 + miR-NC or circ_0003074 + miR-516b-5p, and cell viability was investigated by CCK-8 (**A**), cell proliferation by EdU assay (**B**), cell apoptosis by flow cytometry analysis (**C**), cell migration by wound-healing assay (**D**), cell invasion by transwell invasion assay (**E**), and the protein expression of PCNA, cleaved caspase-3 and MMP9 by Western blotting (**F**, **G**). *P < 0.05, **P < 0.01 and ***P < 0.001

In this part, we transfected circ_0003074, circ_0003074 + miR-516b-5p or matched controls into Rg3-treated MG63 cells and Saos2 cells to explore whether circ_0003074 functioned through miR-516b-5p. As expected, circ_0003074 overexpression promoted cell viability and cell proliferation but inhibited cell apoptosis, whereas these effects were attenuated when miR-516b-5p was increased (Fig. 5A–C). Consistently, the increased cell migration and invasion by enforced expression of circ_0003074 were relieved after miR-516b-5p upregulation (Fig. 5D and E). In support, ectopic circ_0003074 expression led to increases of PCNA and MMP9 but a decrease of cleaved caspase-3; however, these effects were remitted after transfection with miR-516b-5p mimics (Fig. 5F and G). Besides, the study showed that circ_0003074 overexpression promoted cell viability, cell proliferation, migration and invasion, accompanied by increases of PCNA and MMP9 and a decrease of cleaved caspase-3; however, these effects were attenuated after miR-516b-5p downregulation (Additional file 2: Figure S2). By the large, all data manifested that the introduction of circ_0003074 increased OS cell malignancy owing to the decrease of miR-516b-5p.

### Circ_0003074 stimulated KPNA4 production by combining with miR-516b-5p in both MG63 cells and Saos2 cells

**Fig. 6:**
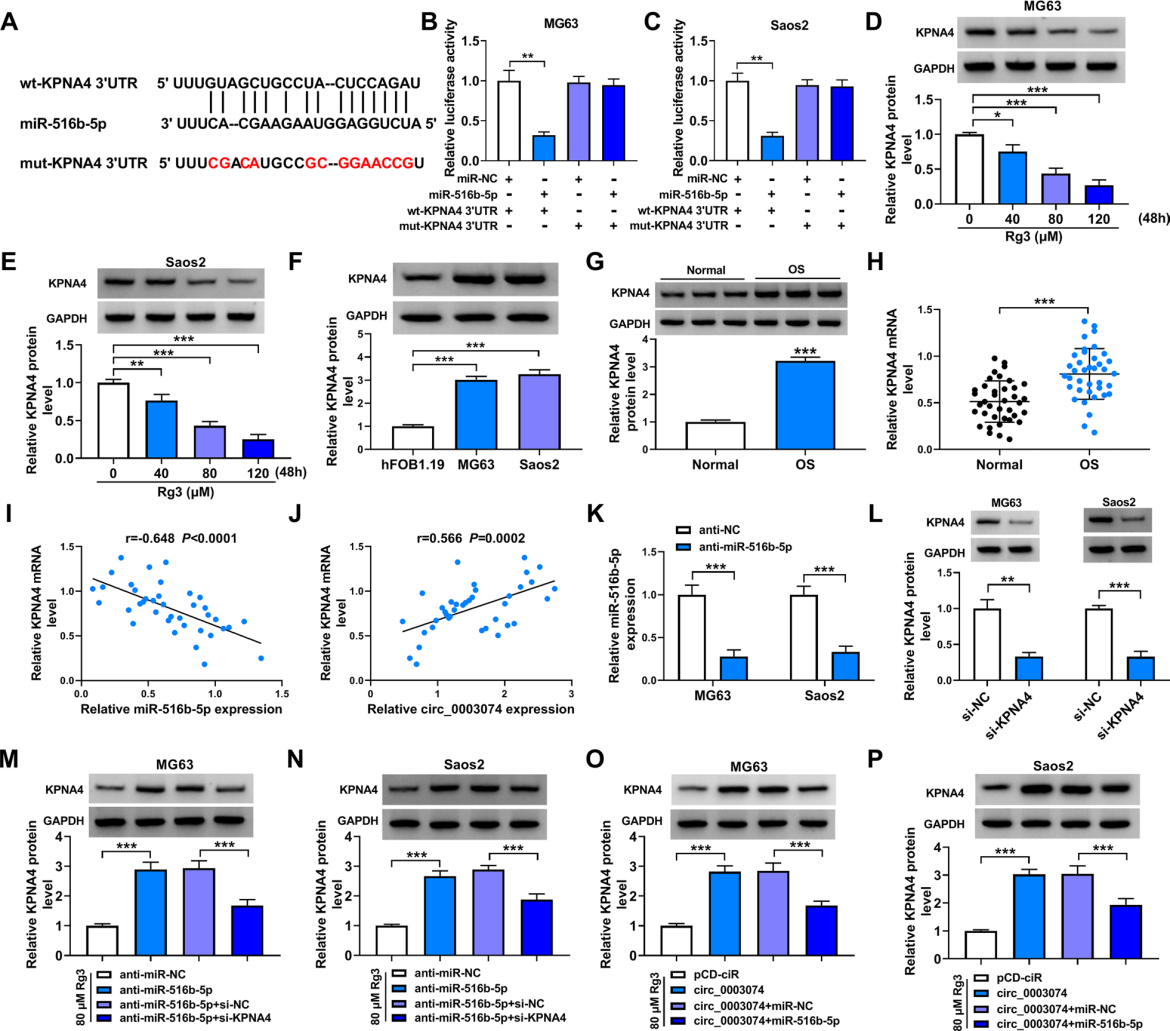
Circ_0003074 regulated KPNA4 expression through miR-516b-5p. **A** The schematic illustration showing the complementary sites of miR-516b-5p with KPNA4. **B**, **C** Dual-luciferase reporter assay was employed to identify the interaction between miR-516b-5p and KPNA4. **D**, **E** KPNA4 protein expression was detected by Western blotting in both MG63 cells and Saos2 cells treated with Rg3 at a dose of 0, 40, 80 or 120 µM. **F**–**H** KPNA4 expression was detected by qRT-PCR and Western blotting in hFOB1.19 cells, MG63 cells, Saos2 cells, OS tissues and adjacent normal tissues. **I**, **J** Spearman correlation analysis was used to confirm the linear correlation between KPNA4 and miR-516b-5p or circ_0003074 in expression in OS tissues. **K**, **L** The efficiency of both miR-516b-5p and KPNA4 knockdown was checked by qRT-PCR or Western blotting in MG63 cells and Saos2 cells. **M**, **N** The effects between miR-516b-5p knockdown and KPNA4 silencing on KPNA4 expression were determined by Western blotting. **O**, **P** Western blotting was carried out to investigate the effects between circ_0003074 overexpression and miR-516b-5p upregulation on KPNA4 protein production. *P < 0.05, **P < 0.01 and ***P < 0.001

Starbase online database was performed to predict the possible target gene of miR-516b-5p. As presented in Fig. 6A, KPNA4, a candidate, carried the binding sites of miR-516b-5p. Subsequently, miR-516b-5p overexpression significantly inhibited the luciferase activity of wt-KPNA4 3ʹUTR, but not that of mut-KPNA4 3ʹUTR (Fig. 6B and C). Based the above results, KPNA4 was employed in the following study. Consistently, Rg3 treatment dose-dependently decreased KPNA4 protein expression in MG63 cells and Saos2 cells (Fig. 6D and E). Comparatively, KPNA4 expression was upregulated in OS cells (MG63 cells and Saos2 cells) and tissues (Fig. 6F–H). When exploring the association between KPNA4 and miR-516b-5p or circ_0003074 in OS tissues, we found that KPNA4 expression was negatively correlated with miR-516b-5p but positively with circ_0003074 (Fig. 6I and J). After determining the successful knockdown of both miR-516b-5p and KPNA4 in MG63 cells and Saos2 cells (Fig. 6K and L), we analyzed the effect of KPNA4 on miR-516b-5p-mediated KPNA4 expression. As shown in Fig. 6M and N, miR-516b-5p absence upregulated KPNA4 protein expression, but the effect was relieved by decreased expression of KPNA4. Furthermore, circ_0003074 overexpression promoted KPNA4 production, which was rescued when miR-516b-5p expression was increased (Fig. 6O and P), showing that circ_0003074 induced KPNA4 production through miR-516b-5p.

### KPNA4 depletion counteracted miR-516b-5p absence-induced OS cell malignancy under Rg3 treatment

**Fig. 7:**
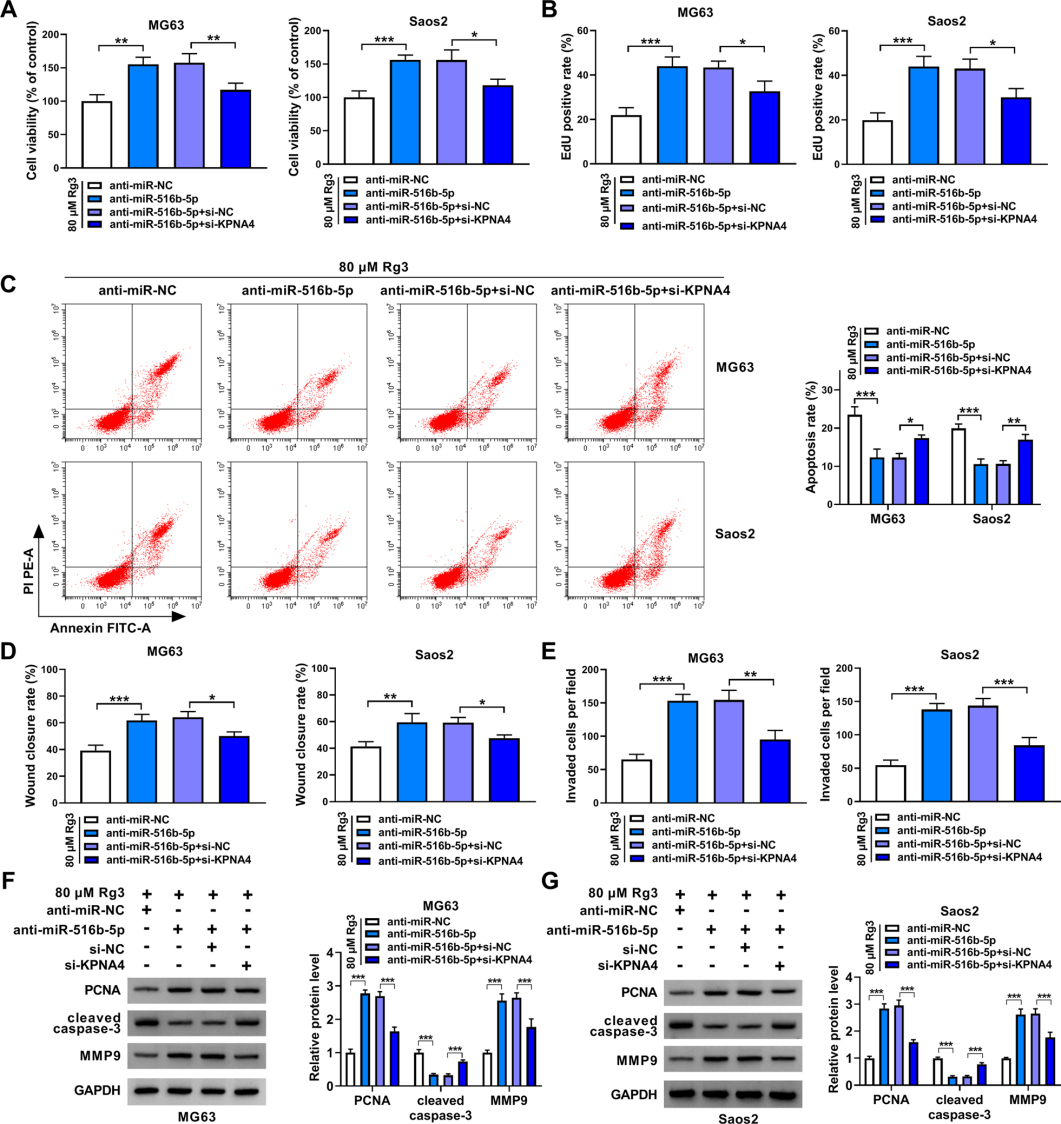
MiR-516b-5p regulated Rg3-treated OS cell malignancy through KPNA4. **A**–**G** Rg3-treated MG63 cells and Saos2 cells were transfected with anti-miR-NC, anti-miR-516b-5p, anti-miR-516b-5p + si-NC, anti-miR-516b-5p + si-KPNA4, and cell viability was investigated by CCK-8 (**A**), cell proliferation by EdU assay (**B**), cell apoptosis by flow cytometry analysis (**C**), cell migration by wound-healing assay (**D**), cell invasion by transwell invasion assay (**E**), and the protein expression of PCNA, cleaved caspase-3 and MMP9 by Western blotting (**F**, **G**). *P < 0.05, **P < 0.01 and ***P < 0.001

Anti-miR-516b-5p, anti-miR-516b-5p + si-KPNA4 or matched controls were transfected into both MG63 cells and Saos2 cells to analyze whether the regulation of miR-516b-5p in Rg3-treated OS cell processes involved KPNA4. MiR-516b-5p silencing enhanced cell viability and promoted cell proliferation, whereas these effects were partly abolished after transfection with si-KPNA4 (Fig. 7A and B). Then, we found that the transfection with miR-516b-5p inhibitors into both MG63 cells and Saos2 cells led to decreased cell apoptosis and increased cell migration and invasion; however, the combined treatment of miR-516b-5p inhibitors and si-KPNA4 rescued these effects (Fig. 7C–E). Besides, decreasing expression of miR-516b-5p promoted PCNA and MMP9 production but inhibited cleaved caspase-3, which was partially restored when knockdown of both miR-516b-5p and KPNA4 (Fig. 7F and G). In support, the above results were confirmed in non-Rg3-treated cells. For instance, miR-516b-5p depletion promoted cell viability, proliferation, migration and invasion, accompanied by increases of PCNA and MMP9 and a decrease of cleaved caspase-3 expression (Additional file 3: Figure S3). Therefore, the above results demonstrated that miR-516b-5p/KPNA4 axis participated in the regulation of Rg3-reduced OS cell malignancy.

### Rg3 treatment inhibited OS cell malignancy in vivo

**Fig. 8:**
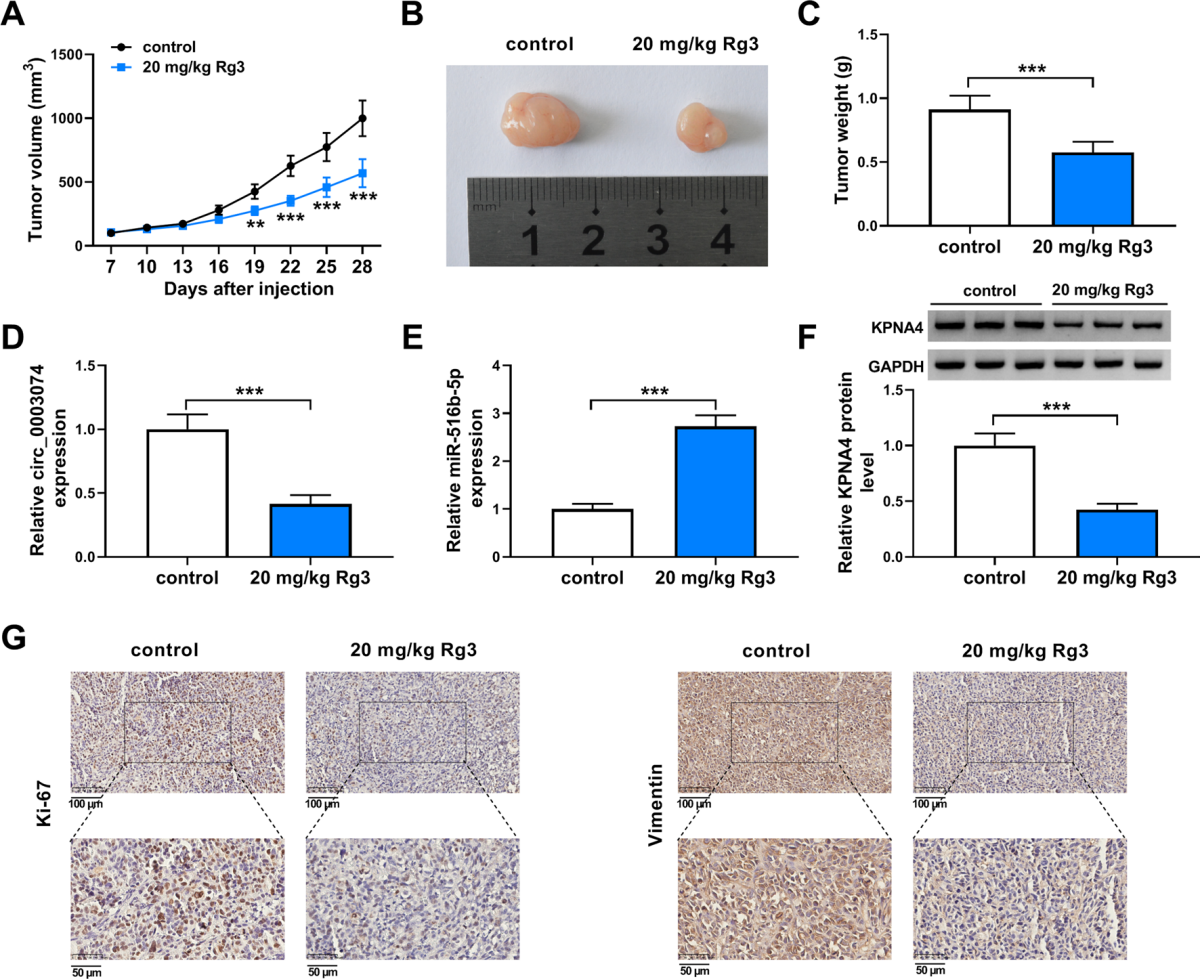
Rg3 treatment inhibited OS cell malignancy in vivo. **A**–**C** The effect of Rg3 treatment on tumorigenesis of MG63 cells. **D**–**F** The impacts of Rg3 treatment on the expression of circ_0003074, miR-516b-5p and KPNA4 were determined by qRT-PCR or Western blotting in the primary tumors from MG63 cells. **G** The positive expression rate of Ki-67 was checked by IHC assay in the primary tumors from MG63 cells. **P < 0.01 and ***P < 0.001

The inhibitory role of Rg3 treatment on OS cell malignancy in vitro was further testified in a nude mouse xenograft. The study found that the mice treated with Rg3 showed significantly decreased tumor volume and weight in comparison with controls (Fig. 8A–C). Comparatively, circ_0003074 and KPNA4 expression were dramatically downregulated, while miR-516b-5p was upregulated in the forming tumors from these mice treated with Rg3 (Fig. 8D–F). Besides, IHC assay exhibited that the positive expression rates of Ki-67 and vimentin were lower in Rg3 treatment group than in control group (Fig. 8G). Taken together, these data manifested that Rg3 inhibited OS cell malignancy by regulating circ_0003074, miR-516b-5p and KPNA4 in vivo.

## Discussion

Rg3 is an active ingredient that is extracted from ginseng and has been confirmed to act an anticancer role in multiple cancers [20]. Lu and his colleagues deciphered that nanoparticles-loaded Rg3 was able to improve photodynamic therapy for OS [9]. Considerable evidences indicated that Rg3 was capacity of inducing cellular apoptosis, attenuating cellular migration and invasion and inhibiting cancer cell resistance to chemotherapy. The inner mechanism of Rg3 in regulating the biological behaviors of cells involves the regulation of miRNA [21], which is the composition of circRNA-miRNA-mRNA [22]. Thus, exploring a circRNA-miRNA-mRNA regulatory axis in revealing the underlying mechanism related to Rg3 in OS progression will be helpful to develop therapeutic strategy for OS. In this work, we demonstrated that Rg3 inhibited OS cell proliferation, migration and invasion but induced all apoptosis in vitro, and reduced tumor growth in vivo. Mechanism assays further proved that Rg3 inhibited OS cell malignancy by circ_0003074/miR-516b-5p/KPNA4 pathway.

CircRNA, discovered in 1970s, is a noncoding RNA that has been confirmed to mediate cancer development. Multiple researches have reported that circRNA is abnormally expressed in OS and participates in OS malignant progression [12]. For instance, circ_0002052 knockdown inhibited OS cell growth and metastasis through interaction with miR-382 [23]. Circ_0005909 silencing reduced tumor growth and restrained cell proliferation and metastasis by inhibiting the production of high mobility group box 1 (HMGB1) in a miR-936-dependent manner [24]. Inversely, circ_0001649 elevation inhibited OS cell survival fraction but induced cell apoptosis through absorbing miR-338-5p, miR-647 and miR-942 [25]. In this work, the roles of circ_0003074 in OS progression and Rg3-mediated OS cell malignancy were revealed for the first time. Herein, circ_0003074 expression was elevated in OS tissues and cells. Rg3 treatment dose-dependently reduced circ_0003074 expression in comparison with controls. Besides, circ_0003074 elevation promoted OS cell proliferation and metastasis but inhibited cell apoptosis. In particular, increased expression of circ_0003074 counteracted Rg3-mediated inhibition of OS cell malignancy.

Given that circRNA was able to function as a miRNA sponge [26], the study then analyzed the target miRNA of circ_0003074. Previous studies have shown that miR-516b-5p is involved in the progression of multiple cancers, which include lung cancer [27], esophageal squamous cell carcinoma [28] and bladder cancer [29]. Herein, miR-516b-5p combined with circ_0003074. Our study further analyzed the function of miR-516b-5p in Rg3-mediated OS cell malignancy. qRT-PCR data showed the downregulation of miR-516b-5p in OS specimen and cell samples. Rg3 treatment dramatically upregulated miR-516b-5p expression in a dose-dependent manner. Besides, miR-516b-5p showed an antagonizing impact against circ_0003074 under Rg3 treatment. Dual-luciferase reporter assay elucidated that miR-516b-5p targeted KPNA4.

KPNA4 is a carrier that mediates multiple transcription factors, and previous data have confirmed that the protein is associated with the localization of Notch intracellular domain [30], which is involved in tumor recurrence [31]. Moreover, it has been shown that KPNA4 regulates the development of prostate cancer [32], glioblastoma [33] and cutaneous squamous cell carcinoma [34]. Besides, Yan et al., suggested that KPNA4, combined with miR-217, contributed to OS cell proliferation and metastasis [35]. In this work, KPNA4 was highly expressed in OS tissues and cells. Rg3 treatment reduced KPNA4 expression. Also, miR-516b-5p inhibition-mediated action was relieved after KPNA4 knockdown. Furthermore, the study confirmed that the promoting impact of circ_0003074 on KPNA4 expression was restored by miR-516b-5p upregulation, which suggested that circ_0003074 induced KPNA4 production through miR-516b-5p.

However, circ_0003074/miR-516b-5p/KPNA4 axis only partially explained Rg3-mediated regulation of OS malignant progression. As a result, it was rational to infer the involvement of other regulators or signaling pathways in Rg3-mediated OS development. In addition, given the lack of genetic characteristics and heterogeneity of tumors derived from xenograft mouse model assay, the in vivo data should be confirmed over a longer period of time using patient-derived tumor xenograft (PDX) model, in vivo assay only testified the role of Rg3 in tumor growth, and further experiments should be designed to confirm the effects of circ_0003074/miR-516b-5p/KPNA4 pathway on Rg3-mediated OS development in vivo.

Taken together, the work revealed a novel signaling pathway, circ_0003074/miR-516b-5p/KPNA4 that weakened the inhibitory impacts of Rg3 on OS cell processes through modulating cell proliferation, metastasis and apoptosis (Additional file 4: Figure S4). Our data show that 80 μM Rg3 or 20 mg/kg Rg3 can significantly inhibit OS cell malignancy. But, the suitable dose of Rg3 used to treat OS still needs more experiments, such as PDX assay, to verify. Besides, there are some problems in applying this compound to treat OS. Firstly, Rg3 affects multiple metabolic pathways, and their efficacy is complex. Secondly, the traditional extraction of RG3 is difficult to be dissolved and the utilization rate is limited. In spite of these limitations, Rg3 is a promising therapeutic agent for OS.

## Supplementary Information

**Additional file 1: Figure S1.** Both MG63 cells and Saos2 cells were treated with Rg3 at a dose of 0, 40, 80, and 120 µM for 24, 48 and 72 h, and cell viability was investigated by CCK-8 (A and B). **P < 0.01 and ***P < 0.001.

**Additional file 2: Figure S2.** Circ_0003074/miR-516b-5p pathway mediated OS cell malignancy. (A-F) Both MG63 and Saos2 cells were transfected with pCD-ciR, circ_0003074, circ_0003074 + miR-NC, circ_0003074 + miR-NC or circ_0003074 + miR-516b-5p, and cell viability was investigated by CCK-8 (A), cell proliferation by EdU assay (B), cell migration by wound-healing assay (C), cell invasion by transwell invasion assay (D), and the protein expression of PCNA, cleaved caspase-3 and MMP9 by Western blotting (E and F). *P < 0.05, **P < 0.01 and ***P < 0.001.

**Additional file 3: Figure S3.** MiR-516b-5p regulated OS cell malignancy through KPNA4. (A-F) MG63 cells and Saos2 cells were transfected with anti-miR-NC, anti-miR-516b-5p, anti-miR-516b-5p + si-NC, or anti-miR-516b-5p + si-KPNA4, and cell viability was investigated by CCK-8 (A), cell proliferation by EdU assay (B), cell migration by wound-healing assay (C), cell invasion by transwell invasion assay (D), and the protein expression of PCNA, cleaved caspase-3 and MMP9 by Western blotting (E and F). *P < 0.05, **P < 0.01 and ***P < 0.001.

**Additional file 4: Figure S4.** The schematic showing the proposed mechanism of the present study.

## Data Availability

Not applicable.

## Abbreviations

OS: Osteosarcoma
Rg3: Ginsenoside Rg3
circRNA: Circular RNA
miRNA: MicroRNA
DHTKD1: Dehydrogenase E1 and transketolase domain containing 1
KPNA4: Karyopherin subunit alpha 4
HMGB1: High mobility group box 1
