# Supplementary figures and images for: Ginsenoside Rg3 inhibits osteosarcoma progression by reducing circ_0003074 expression in a miR-516b-5p/KPNA4-dependent manner
Source: J Orthop Surg Res. 2021 Dec 20;16:724. doi: 10.1186/s13018-021-02868-7 (PMC8686618; doi:10.1186/s13018-021-02868-7)

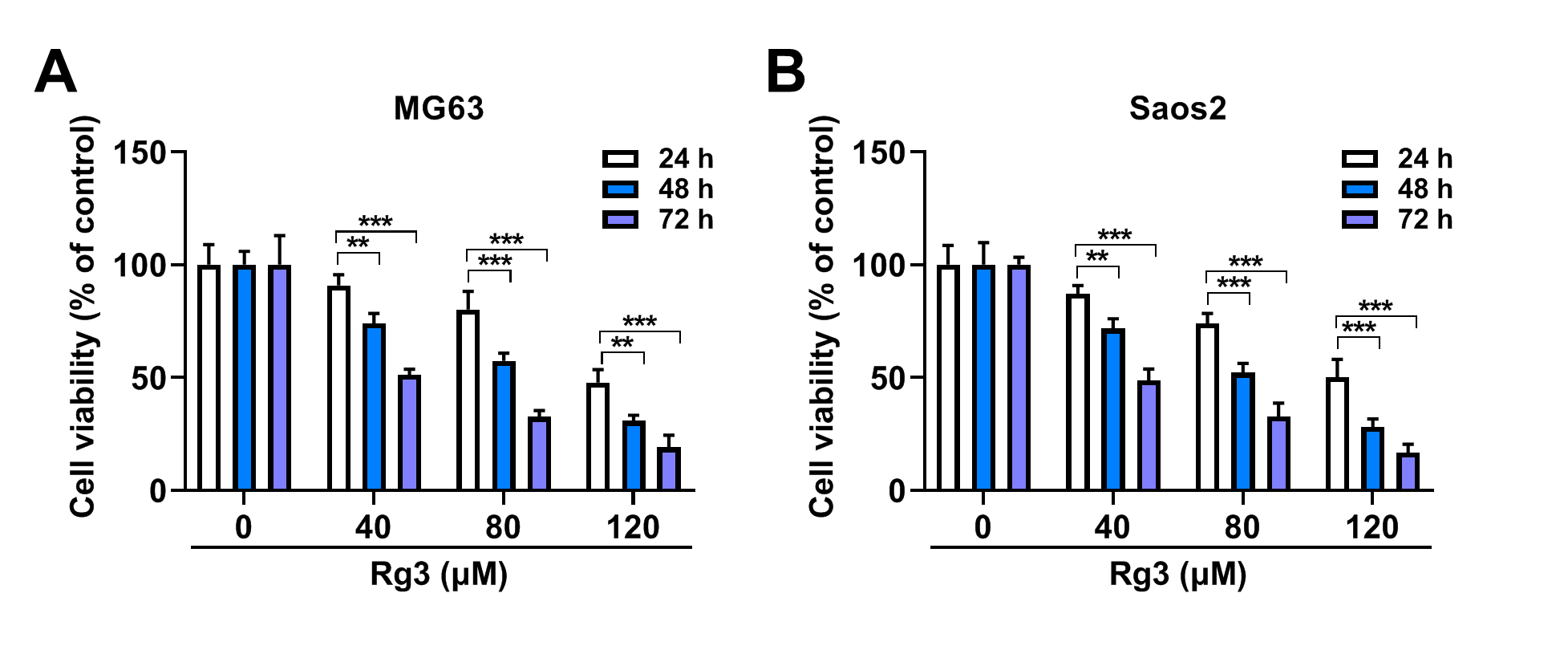

Supplement: Supplementary file 1 — Additional file 1: Figure S1. Both MG63 cells and Saos2 cells were treated with Rg3 at a dose of 0, 40, 80, and 120 µM for 24, 48 and 72 h, and cell viability was investigated by CCK-8 (A and B). **P < 0.01 and ***P < 0.001. [file 13018_2021_2868_MOESM1_ESM.tif]

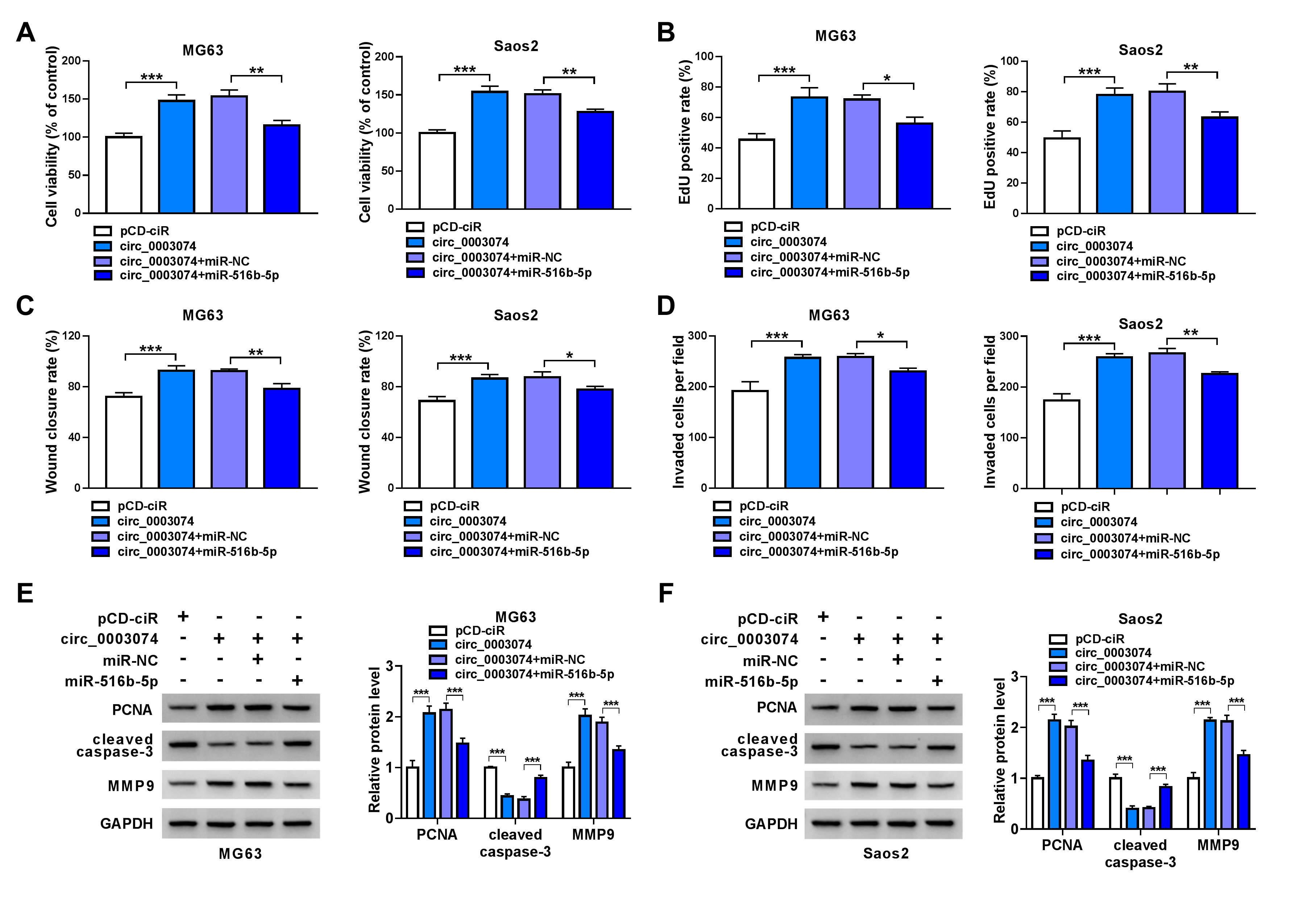

Supplement: Supplementary file 2 — Additional file 2: Figure S2. Circ_0003074/miR-516b-5p pathway mediated OS cell malignancy. (A-F) Both MG63 and Saos2 cells were transfected with pCD-ciR, circ_0003074, circ_0003074 + miR-NC, circ_0003074 + miR-NC or circ_0003074 + miR-516b-5p, and cell viability was investigated by CCK-8 (A), cell proliferation by EdU assay (B), cell migration by wound-healing assay (C), cell invasion by transwell invasion assay (D), and the protein expression of PCNA, cleaved caspase-3 and MMP9 by Western blotting (E and F). *P < 0.05, **P < 0.01 and ***P < 0.001. [file 13018_2021_2868_MOESM2_ESM.tif]

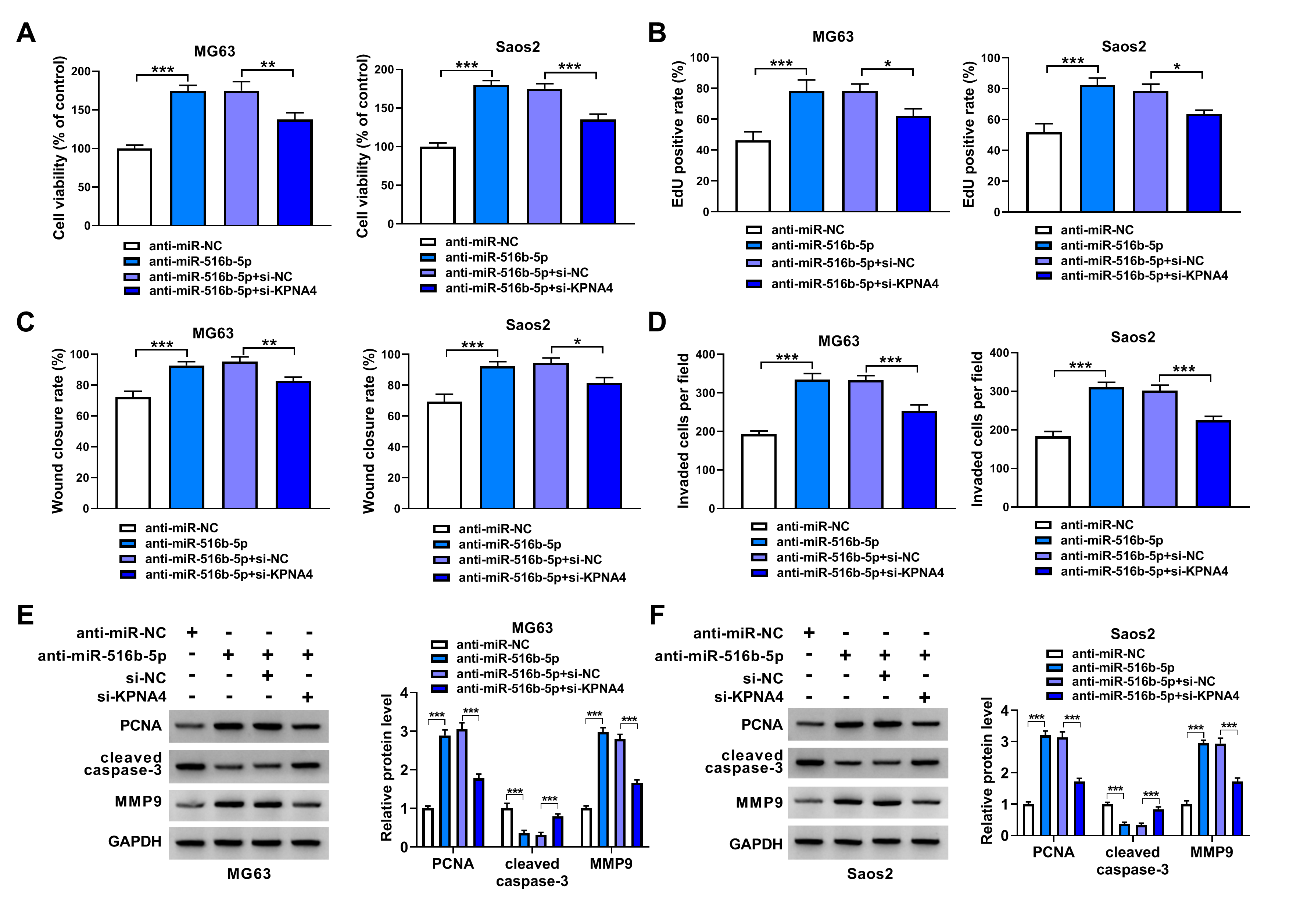

Supplement: Supplementary file 3 — Additional file 3: Figure S3. MiR-516b-5p regulated OS cell malignancy through KPNA4. (A-F) MG63 cells and Saos2 cells were transfected with anti-miR-NC, anti-miR-516b-5p, anti-miR-516b-5p + si-NC, or anti-miR-516b-5p + si-KPNA4, and cell viability was investigated by CCK-8 (A), cell proliferation by EdU assay (B), cell migration by wound-healing assay (C), cell invasion by transwell invasion assay (D), and the protein expression of PCNA, cleaved caspase-3 and MMP9 by Western blotting (E and F). *P < 0.05, **P < 0.01 and ***P < 0.001. [file 13018_2021_2868_MOESM3_ESM.tif]

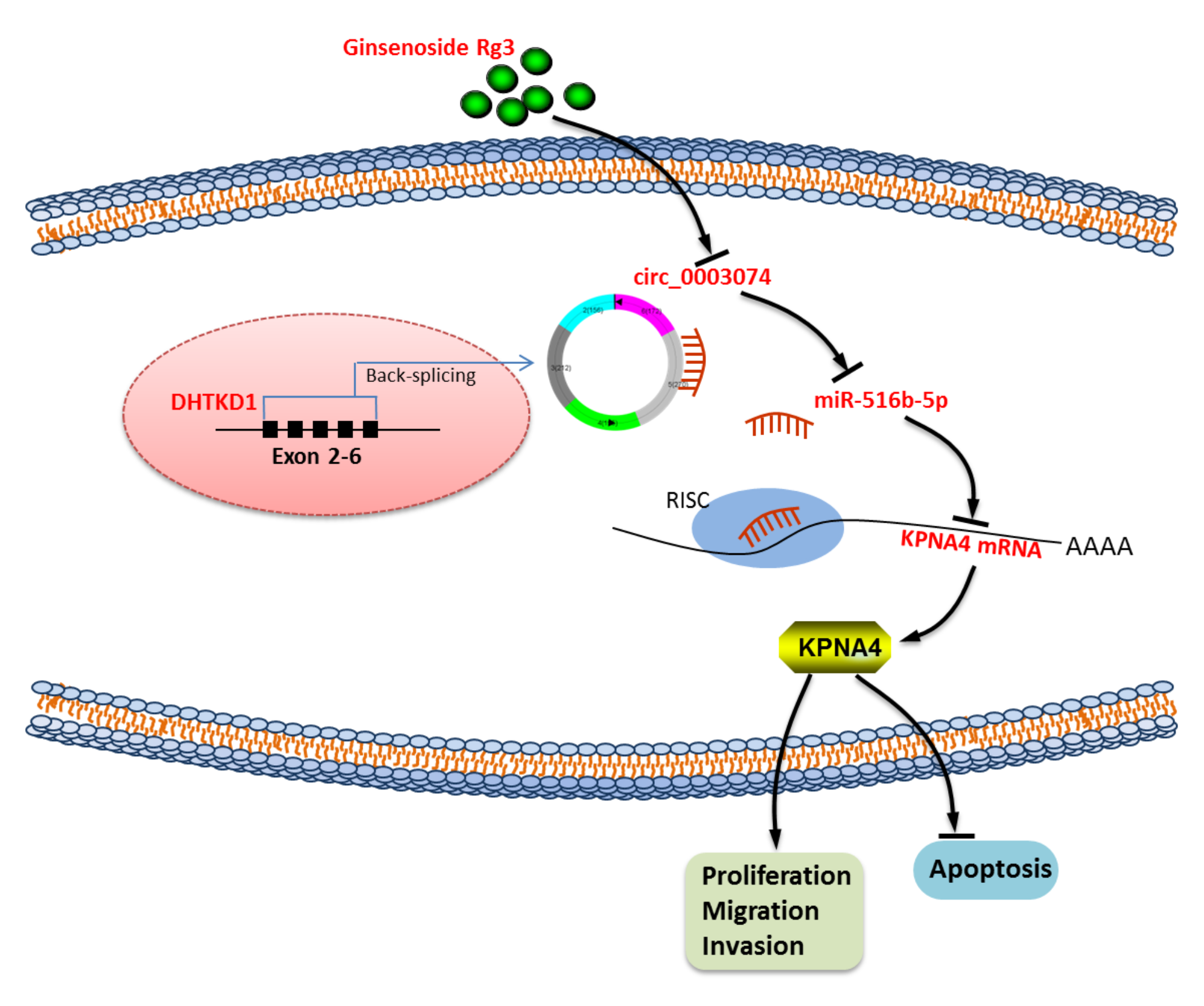

Supplement: Supplementary file 4 — Additional file 4: Figure S4. The schematic showing the proposed mechanism of the present study. [file 13018_2021_2868_MOESM4_ESM.tif]
